# Spectroscopic Insights into the Localization and Photodynamic Efficacy of Aluminum Tetrasulfonated Phthalocyanine for Colorectal Cancer Therapy

**DOI:** 10.1021/acs.jpcb.5c01189

**Published:** 2025-08-12

**Authors:** K. Beton-Mysur, A. Jarota, M. Wolszczak, B. Brozek-Pluska

**Affiliations:** † Laboratory of Laser Molecular Spectroscopy, Institute of Applied Radiation Chemistry, Faculty of Chemistry 49584 , Lodz University of Technology, Wroblewskiego 15, 93-590 Lodz, Poland; ‡ Institute of Applied Radiation Chemistry, Faculty of Chemistry, Lodz University of Technology, Wroblewskiego 15, 93-590 Lodz, Poland

## Abstract

This article explores the potential of aluminum tetrasulfonated phthalocyanine (AlPcS_4_) as a photosensitizer for photodynamic therapy (PDT) in colorectal cancer (CRC), utilizing Raman imaging, steady-state absorption and fluorescence spectroscopy in UV–Vis spectral region, and transient absorption spectroscopy. Our study demonstrates that in human colon cancer cells, the administered photosensitizer preferentially localizes to the endoplasmic reticulum and lipid droplets, mirroring its distribution in normal cells. Furthermore, the addition of DTAC significantly enhances the permeability of cell membranes to AlPcS_4_, leading to an increased intracellular concentration of the photosensitizer, as evidenced by the elevated fluorescence intensity around 679 nm, even after just 30 min of incubation. Photochemical property assessments of AlPcS_4_ in both cellular and cell-free environments indicate only minimal interaction with cells. The differences observed in absorption and fluorescence spectra, as well as in the singlet excited state lifetime of AlPcS_4_ in the presence of cells, are negligible compared to those measured in a neat buffer solution. However, the extended triplet-state lifetime observed with both Caco-2 and CCD-18Co cells (460 μs) versus buffer alone (407 μs) provides clear evidence of interaction between AlPcS_4_ and the cells.

## Graphical abstract

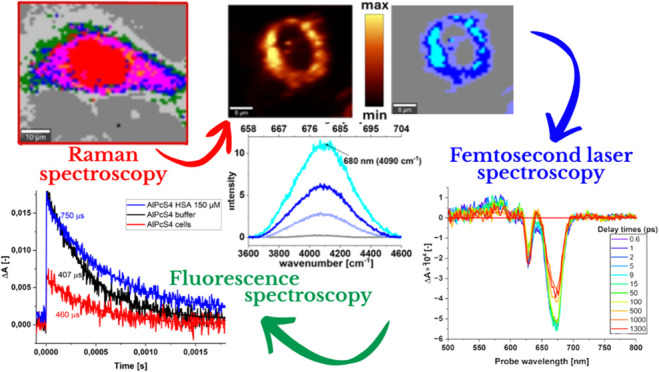

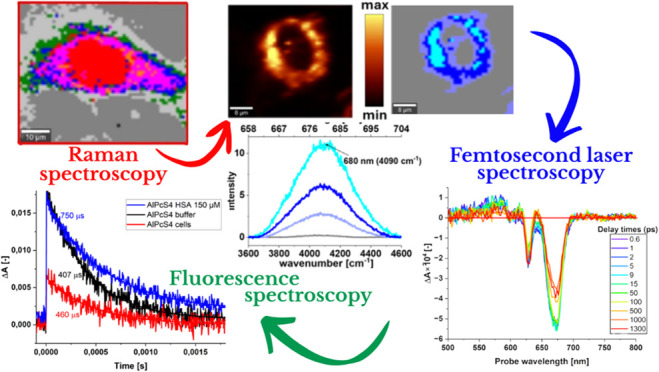

## Introduction

Colorectal cancer (CRC) poses a major global health challenge, ranking as the second leading cause of cancer-related deaths worldwide. Over the past decade, CRC incidence rates have been steadily increasing, with estimates suggesting that in 2020, CRC accounted for approximately 10% of all cancer cases worldwide.^1^ Standard therapeutic approaches for colorectal cancer (CRC), including surgical resection, chemotherapy, and radiotherapy, remain the cornerstone of clinical management. However, these modalities frequently fail to achieve complete remission due to their invasive nature, systemic toxicity, and associated adverse effects. This highlights the critical need for the development of novel and more efficacious therapeutic strategies that can enhance treatment outcomes while minimizing undesirable side effects.

Photodynamic therapy (PDT) emerges as a promising antitumor approach for CRC, offering a less detrimental option that selectively targets cancer cells with minimal adverse effects.^2–4^ Moreover, PDT is well-tolerated for repeated dosing and demonstrates greater efficacy compared to traditional CRC treatments.^5,6^ Furthermore, photodynamic therapy (PDT) is well-tolerated upon repeated administration and exhibits superior therapeutic efficacy in comparison to conventional treatments for colorectal cancer (CRC).^5,6^ In addition, photodynamic protocols are noninvasive and painless, with procedural simplicity that facilitates their application in outpatient settings. PDT also holds therapeutic potential in the management of chronic inflammatory conditions and serves as an alternative approach for treating drug-resistant bacterial infections.^7^

Among the extensively investigated metal phthalocyanines (MPcs), tetrasulfonated aluminum phthalocyanine (AlPcS_4_) has demonstrated utility as a photosensitizing agent in the treatment of various solid malignancies, including breast, colorectal, and esophageal cancers.^8–13^ The molecular structure of AlPcS_4_ is depicted in Figure 1A.

**Figure 1:**
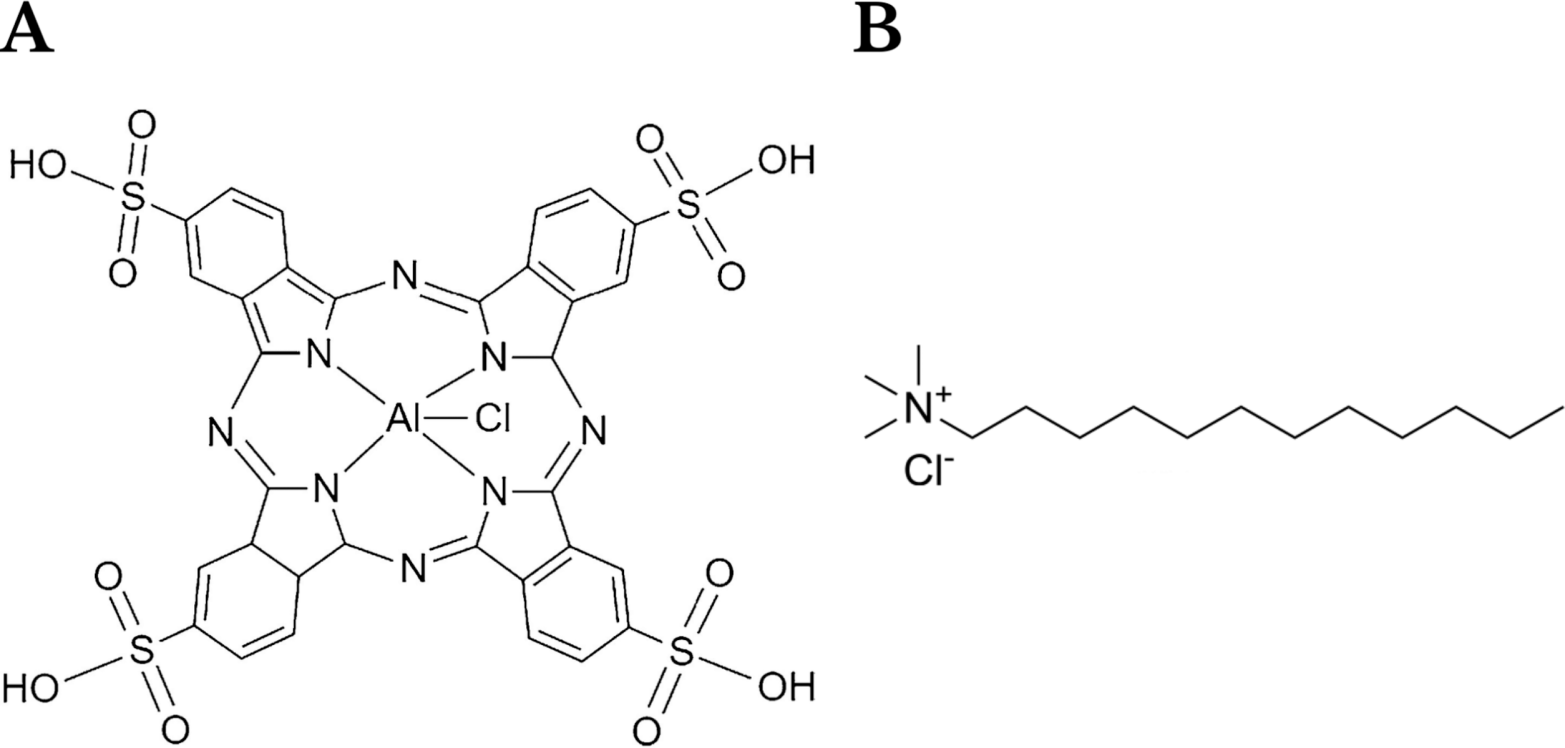
Structural formula of tetrasulfonated aluminum phthalocyanine (AlPcS_4_) (panel A) and dodecyl trimethylammonium chloride (DTAC) (panel B).

Phthalocyanines, including AlPcS_4_, are among the most extensively studied and well-characterized photosensitizers. They exhibit light absorption within a spectral range comparable to that of porphyrins and primarily exert their photodynamic effects through a Type II photosensitization mechanism, which entails the generation of singlet oxygen - a critical cytotoxic species responsible for the destruction of tumor cells.

The presence of sulfonate groups in AlPcS_4_, in contrast to nonsulfonated derivatives, enhances its water solubility without significantly impairing its photophysical properties. In addition, AlPcS_4_ does not form aggregates in water in a wide range of concentration (10^–7^–10^–4^ M) in aqueous solution, primarily due to the presence of the negatively charged sulfonate group**s** on the phthalocyanine ring.^14^ The axial ligand, the chloride ion, can also play a role in stabilizing the individual molecules. The lack of tendency to aggregate makes AlPcS_4_ a great candidate for use as a PS in PDT, as only monomers are effective in this therapy. AlPcS_4_ exhibits robust absorption of visible light within the wavelength range of 650–800 nm, optimizing tissue penetration, a critical factor for PDT. The molar absorption coefficient for AlPcS_4_ is as high as 1.7 × 10^5^ M^–1^ cm^–1^ at 675 nm,^15^ allowing for lower optical power to achieve therapeutic effects compared to porphyrin-based photosensitizers. This feature enables effective cancer tissue treatment with minimal phototoxicity. Prior *in vitro* investigations employing AlPcS_4_ PS agents have been documented.^16,17^ Chizenga et al. observed a significant dose-dependent reduction in cell proliferation and heightened cytotoxicity in cervical cancer cells and cervical Cancer Stem Cells (CSCs) following irradiation with a 673.2 nm diode laser.^16^ Additionally, AlPcS_4_-induced PDT has been shown to markedly augment singlet oxygen quantum yields and proficiently disrupt cell membranes and proteins.^17^

A widely adopted strategy to enhance the cellular uptake of photosensitizers (PS) and improve their targeted delivery to malignant cells involves the use of auxiliary carrier systems. Commonly employed carriers for this purpose include liposomes, polymeric nanoparticles, dendrimers, nanocapsules, and other nanoscale delivery platforms. In the present study, we evaluated the efficacy of dodecyl trimethylammonium chloride (DTAC) micelles in enhancing the cellular uptake and functional properties of AlPcS_4_. The chemical structure of DTAC is illustrated in Figure 1B, while the conceptual framework depicting the role of micellar nanocarriers in cancer therapy is presented in Figure 2.

**Figure 2:**
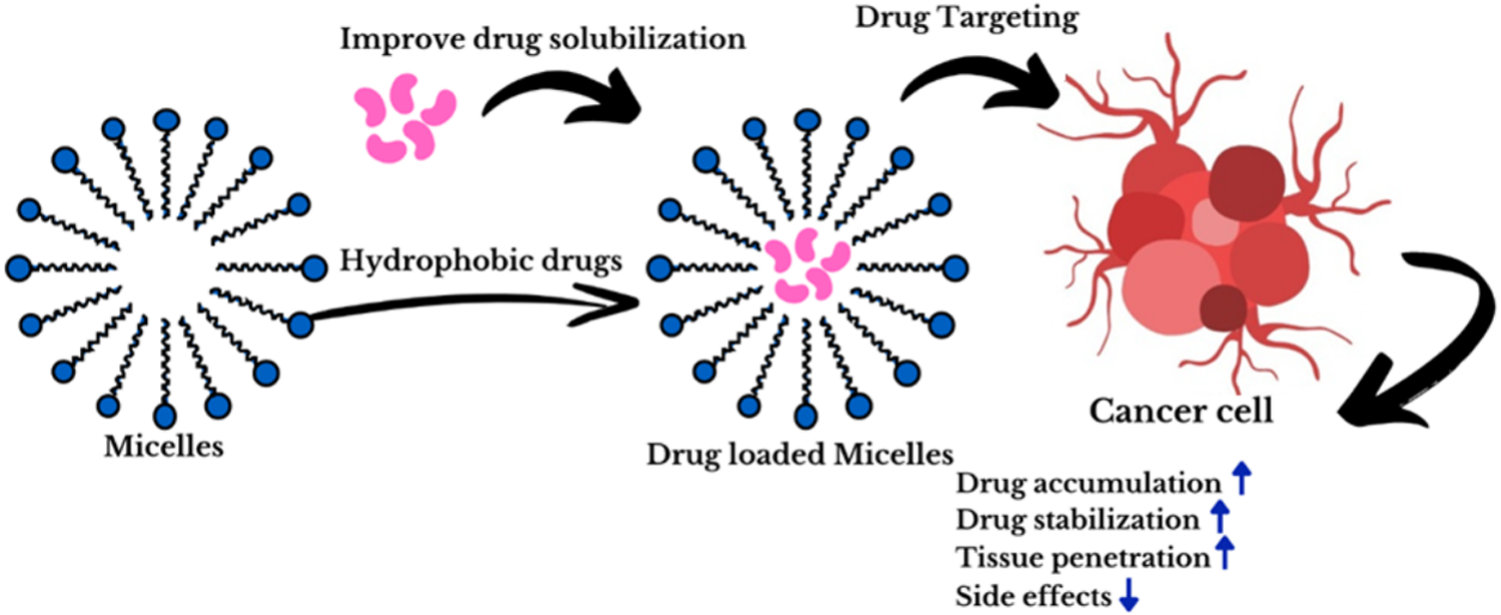
Schematic presentation of micelles nanocarriers impact on cancer therapy.

Once the concentration of surfactants reaches a critical micelle concentration (CMC), they form micelles – structured self-aggregated units acting like flexible and nonspecific hosts often referred to as ″soft cages″. These micelles have precisely defined sizes and beneficial optical characteristics, making them valuable as models for cell membranes. Furthermore, micelles hold promise as drug delivery systems for PDT due to their controllable properties and favorable pharmacological attributes.^18,19^ The DTAC micelles are also often utilized in research settings for various applications, including drug delivery and as models for cell membranes.^20^

This study investigates the potential of AlPcS_4_ in photodynamic therapy, with particular emphasis on its intracellular localization and selective accumulation in normal and cancerous human colon cells, as assessed by Raman imaging in the presence and absence of DTAC micelles. Additionally, the influence of cellular environments and micellar carriers on the UV–vis absorption, fluorescence spectra, and relaxation dynamics of AlPcS_4_ was examined. The findings underscore the utility of Raman imaging, absorption and fluorescence spectroscopy, as well as transient absorption techniques, in characterizing the distribution and photophysical behavior of photosensitizers.

## Materials and Methods

### Cell Lines and Cell Culture

CCD-18Co cell line (ATCC CRL-1459) was purchased from ATCC: The Global Bioresource Center. CCD-18Co cell line was cultured using ATCC-formulated Eagle’s Minimum Essential Medium with l-glutamine (catalog No. 30-2003). To make the complete growth medium, fetal bovine serum was added to a final concentration of 10%. Every 2–3 days, a new medium was used. The cells obtained from the patient are normal myofibroblasts in the colon. The biological safety of the CCD-18Co cell line has been classified by the American Biosafety Association (ABSA) as level 1 (BSL-1). The Caco-2 cell line was also purchased from ATCC and cultured according to the ATCC protocols. The Caco-2 cell line was obtained from a patient - a 72-year-old Caucasian male diagnosed with colon adenocarcinoma. The biological safety of the obtained material is classified as level 1 (BSL-1). To make the medium complete, we based on Eagle’s Minimum Essential Medium with l-glutamine, with the addition of a fetal bovine serum to a final concentration of 20%. The medium was renewed once or twice a week.

### Cultivation Conditions

Cell lines (CCD-18Co, Caco-2) used in the experiments in this study were grown in flat-bottom culture flasks made of polystyrene with a cell growth surface of 75 cm^2^. Flasks containing cells were stored in an incubator providing environmental conditions at 37 °C, 5% CO_2_, 95% air.

### Sample Preparation

Tetrasulfonated aluminum phthalocyanine stock solutions were prepared in Millipore water and kept at approximately 7 °C under shielded conditions from ambient light. The purity and concentration of these solutions were verified using UV–visible spectroscopy. Detergent solutions were prepared by adding water to a measured amount of DTAC. The required volume of AlPcS_4_ stock solution was thoroughly dissolved in the DTAC solution to achieve a final concentration of 10 μM AlPcS_4_.

### Raman Imaging, Fluorescence Data

All maps and Raman spectra presented and discussed in this paper were recorded using the confocal microscope α 300 RSA+ (WITec, Ulm, Germany) equipped with an Olympus microscope integrated with fiber with 50 μm core diameter with a UHTS spectrometer (Ultra High Through Spectrometer) and a CCD Andor Newton DU970NUVB-353 camera operating in default mode at −60 °C in full vertical binning mode. 532 nm excitation laser line, which is the second harmonic of the Nd: YAG laser, was focused on the sample through a Nikon objective lens with a magnification of 40x and a numerical aperture (NA = 1.0) intended for cell measurements performed by immersion in PBS. The average excitation power of the laser during the experiments was 10 mW, with an integration time of 0.3 s for Raman measurements for the high-frequency region and 0.5 s for the low-frequency region. An edge filter was used to filter out the Rayleigh scattered light. A piezoelectric table was applied to set the test sample in the right place by manipulating the XYZ positions and consequently recording Raman images. Spectra were acquired with one acquisition per pixel and a diffraction grating of 1200 lines/mm. Cosmic rays were removed from each Raman spectrum (model: filter size: 2, dynamic factor: 10), and the Savitzky-Golay method was implemented for the smoothing procedure (order: 4, derivative: 0). All data were collected and processed using special original software WITec Project Plus. All imaging data were analyzed by Cluster Analysis (CA), which allows for the grouping of a set of vibrational spectra that bear resemblance to each other. CA was executed using WITec Project Plus software with a Centroid model and k-means algorithm, in which each cluster is represented by one vector of the mean.

An Alpha 300 RSA+ confocal microscope was also used for fluorescence data acquisition. The fluorescence spectra were recorded in a spectral range 4100 cm^–1^ with an integration time: 0.01 s.

### Femtosecond Laser System

We have conducted experiments using transient absorption (TA) techniques employing an ultrafast laser system. The system comprises a femtosecond laser oscillator (Tsunami, Spectra-Physics, 82 MHz, 800 nm, pulse duration less than 100 fs). The oscillator is pumped by a diode laser (Millennia Pro, Spectra-Physics, 532 nm, 5 W). The laser pulses generated by the oscillator are subsequently amplified in a regenerative amplifier (Spitfire ACE, Spectra-Physics, 1 kHz, output power: 4 W). The amplified pulses seed two optical parametric amplifiers (OPA, Topas Prime, Light Conversion) that are used as a source of pump and probe pulses in transient absorption measurement. The pulse duration at the sample position was ∼120 fs as determined by cross-correlation between pump and probe pulses. The energies of pump and probe pulses in TA experiments were 150 and 15 nJ, respectively.

The Δ*A* signal was recorded by a detection system consisting of a monochromator (iHR320) equipped with a photomultiplier (PMTSS, Thorlabs). The pump and probe pulses were used directly from OPAs. The polarization between pump and probe pulses was adjusted to a magic angle (∼54.6°) to reduce contributions from molecular reorientations to the Δ*A* signal.

### Fluorescence Measurements

Details of steady-state fluorescence and absorbance measurements, as well as time-resolved absorbance and fluorescence detection methods in the nanosecond time domain, are given in ref 21. In flash photolysis measurements, the GL-3300 nitrogen laser (Photon Technology International) was used instead of the Lambda-Physik COMPex 201 excimer laser.

### Data Normalization

The normalization, model: divided by norm (divide the spectrum by the data set norm) was performed by using Origin software according to the formula:

$$V^{\prime }=\frac{V}{∥V∥}$$

$$∥V∥=\sqrt{{v_{1}}^{2}+{v_{2}}^{2}+...+{v_{n}}^{2}}$$

where *n* is the *n*th *V* value.

The normalization was performed for all Raman spectra presented in the manuscript.

### Chemical Compounds

Tetrasulfonated aluminum phthalocyanine (AlPcS_4_), Catalogue Number: 362530-1G, was purchased from Merck Life Science Sp. z o. o., and used without additional purification. DTAC from Eastman Kodak Co. was recrystallized twice from a mixture of 10% ethanol in acetone. All the substances were used without further purification. Super clean distilled water was purified with the Millipore Milli-Q system.

## Results and Discussion

Raman spectroscopy (RS) and Raman imaging (RI), both based on the principle of inelastic light scattering, were employed to obtain detailed insights into the vibrational characteristics and chemical composition of the analyzed samples..^22–24^

Additionally, the mapping mode enables the determination of the spatial distribution of various compounds within the sample, including key biological building blocks such as proteins, lipids, saccharides, and water..^25–28^ Moreover, we have employed confocal Raman imaging due to its high lateral and axial resolution to look inside their organelles without having to destroy the cells’ integrity.^28–30^

Nowadays, RI is a valuable tool for single-cell spectroscopic analysis based on molecule vibrations.^31–33^ Scheme 1 illustrates the conceptual framework of Raman imaging (RI) measurements and highlights the multiplexing capabilities of Raman scattering (RS) and RI, whereby a single acquisition enables the simultaneous detection of multiple spectral features, yielding detailed and comprehensive information on the chemical composition of the sample.

**Scheme 1:**
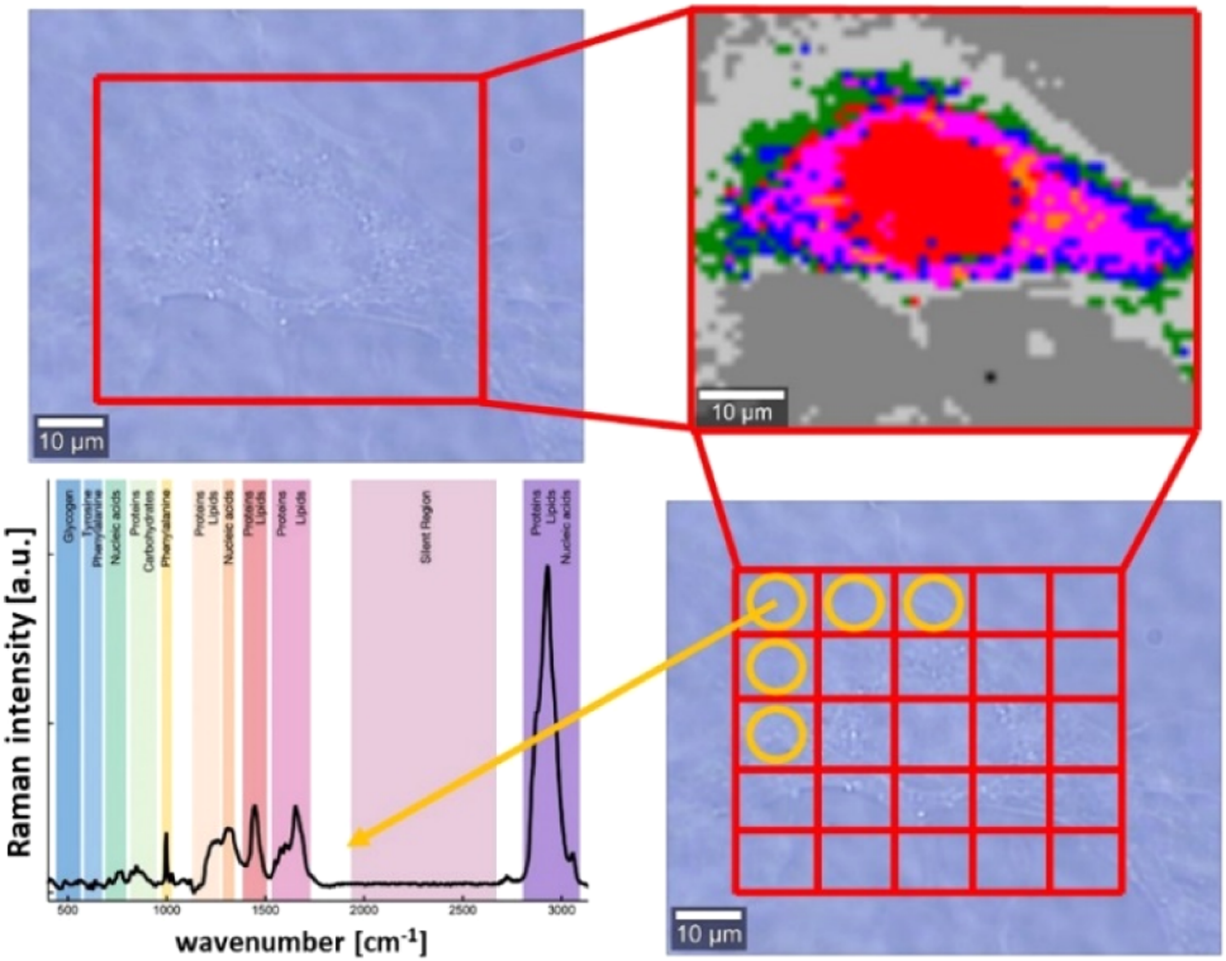
Schematic Representation of the RI Measurements Idea and Explanation of RS and RI Multiplexing Capabilities^a^

a Spectral interpretation based on ref 22.^22^

In our previous studies, we demonstrated that Raman imaging (RI) in combination with cluster analysis (CA) algorithms enables effective visualization and chemical characterization of subcellular organelles, including the nucleus, mitochondria, endoplasmic reticulum (ER), lipid droplets (LDs), cytoplasm, and cell membrane.^31,34–37^ This methodological approach is particularly advantageous for investigating the selective accumulation of photosensitizers (PS) within individual cells.

Figure 3 and Table 1 show the average Raman spectra of the above-mentioned organelles for single normal and cancerous human colon cells in the fingerprint region and the tentative assignment of the Raman peaks marked in the Raman spectra for the fingerprint region: 500–1900 cm^–1^.^22^

**Figure 3:**
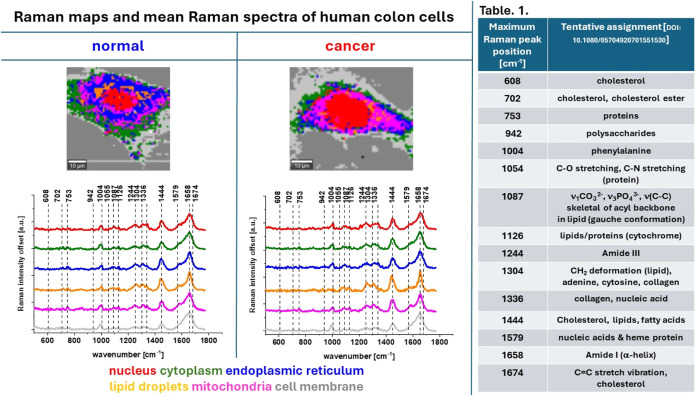
Raman maps and mean Raman spectra of all organelles of normal and cancer human colon single cells identified using CA: nucleus (red), mitochondria (magenta), ER (blue), LDs (orange), cytoplasm (green), cell membrane (light gray). Table 1 Tentative assignment of Raman peaks marked on Raman spectra.^22^

As shown in Figure 3 and Table 1, RS and RI enable the identification of a wide range of chemical compounds, including nucleic acids, proteins, lipids, cholesterol derivatives, and saccharides - each playing essential roles in genetic information storage, cell function, energy metabolism, hormone regulation, and interorgan communication. To compare objectively the biochemical composition of human normal and cancer colon cells, we have performed chemometric analysis in the form of Principal Component Analysis (PCA). PCA allows us to determine the significant differences between the analyzed cell types.^38–40^ Figure 4 shows the results of PCA-loading plots for human normal and cancer colon cells without any supplementation.

**Figure 4:**
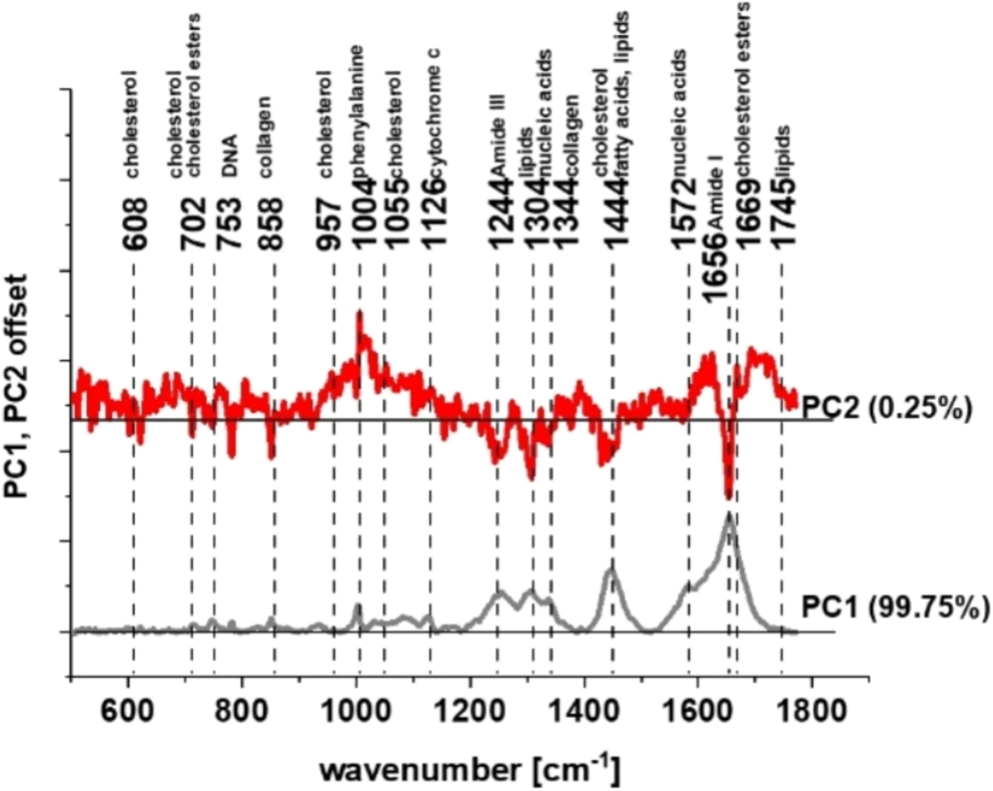
PCA loading plots for human normal and cancer colon cells without any supplementation (legend: PC1 – principal component no. 1 and PC2 – principal component no. 2).

Figure 4 shows that the spectral profiles of the two human colon cell lines - CCD-18Co (normal) and Caco-2 (cancer) – differ significantly at several Raman bands: 1004, 1244, 1304, 1444, 1656, 1659, and 1745 cm^–1^. It indicates that differences between colon cells are found for Raman peaks typical for proteins, nucleic acids, lipids, sterols (including cholesterol), and saccharides.

Based on the Raman data and chemometric analysis, we conclude that the bands differentiating normal and cancer human colorectal cells are characteristic of the main macromolecular components and that two principal components account for the entire variance.

This result is expected, as cancer cells typically exhibit protein overexpression, altered lipid metabolism, and an increased demand for saccharides..^41–43^

A similar RI analysis was conducted for normal and cancerous human colon cells following AlPcS_4_ supplementation. Figure 5A shows the Raman imaging, mean Raman spectra of all organelles identified by CA: nucleus (red), mitochondria (magenta), ER (blue), LDs (orange), cytoplasm (green), and cell membrane (light gray). The filter at 2854 cm^–1^ is widely used in Raman spectroscopy, particularly in imaging biological structures, due to its specificity for symmetric stretching vibrations of C–H bonds, which are characteristic of lipids. This spectrum enables precise imaging of lipid-rich structures and cell membranes, due to the high Raman signal intensity from −CH_2_ and −CH_3_ stretching vibrations.

**Figure 5:**
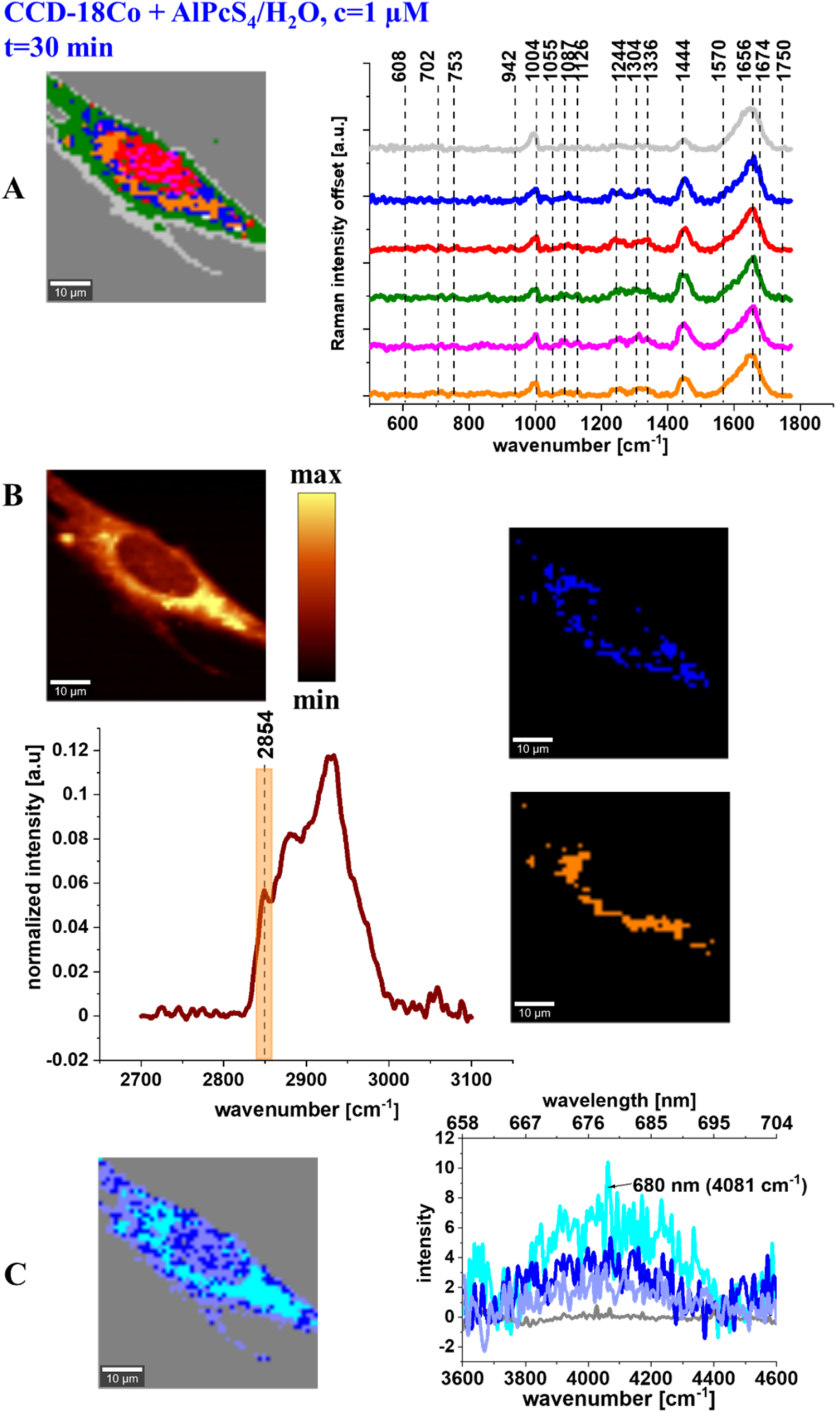
Raman imaging and mean Raman spectra of all organelles identified by CA: nucleus (red), mitochondria (magenta), ER (blue), LDs (orange), cytoplasm (green), cell membrane (light gray) (A), filter showing lipids distribution based on Raman peak centered at 2854 cm^–1^, mean spectrum of the cell as a whole in the high-frequency range and the filter range marked in orange, maps of ER (blue) and LDs (orange) obtained based on CA (B), and Raman imaging of AlPcS_4_ distribution based on CA algorithm and fluorescence spectra of photosensitizer for normal human colon cells – CCD-18Co, for 30 min supplementation of the phthalocyanine (C), the colors of the spectra correspond to the colors of Raman maps.

In our study, this approach enabled precise visualization of lipid alterations between normal and cancerous colon cells. In practice, RI utilizing the 2854 cm^–1^ filter is highly valuable in medical diagnostics, particularly in cancer research and metabolic disease studies, where lipid distribution plays a key role in pathogenesis.^44–47^ Compared to other imaging techniques, RS using this filter offers high chemical specificity without the need for dyes or fluorescent markers, making it a powerful tool in modern biomedical research. Panel 5B presents a lipid distribution map based on the Raman peak centered at 2854 cm^–1^, the mean Raman spectrum in the high-frequency region for the cell as a whole, and maps of ER and LDs obtained based on CA. Panel 5C shows the Raman imaging of PC distribution based on the CA algorithm, along with corresponding fluorescence spectra of AlPcS_4_ for normal human colon cells - CCD-18Co, after 30 min of photosensitizer supplementation. The colors of the spectra match the colors used in the Raman maps.

Figure 5 shows that AlPcS_4_ supplementation provides complex information about the chemical composition of the cells. Well-resolved Raman peaks are observed and can be assigned to all the types of chemical compounds discussed above for cells cultured without photosensitizer (see Figure 3, panel normal). Moreover, fluorescence spectra of AlPcS_4_ recorded using a Raman spectrometer provide information about the photosensitizer distribution and relative concentration. Figure 5, panels B and C, show that upon AlPcS_4_ supplementation, the photosensitizer preferentially localizes in lipid-rich structures such as ER (blue Raman map) and LDs (orange Raman map). This observation is further confirmed by the Raman filter centered at 2854 cm^–1^, the frequency typical for lipids.^22,48,49^

A perfect match can be observed between the maps presented in panel B, regardless of whether they were generated using the filter mode or CA. Additionally, the Raman map created using CA and based on fluorescence spectra typical for AlPcS_4_ (centered at ca. 679 nm) presented in panel C further confirms again that the photosensitizer preferentially localizes in lipid substructures, with the highest intensity of fluorescence once again observed for ER and LDs (turquois and blue spectra, respectively).

We extended our research on normal human colon cells by increasing the supplementation time to 24 h to investigate potential time-dependent effects. Figure 6 shows the results of our analysis, including Raman maps, mean Raman spectra, a Raman filter illustrating the lipid distribution, and maps of ER and LDs generated using CA.

**Figure 6:**
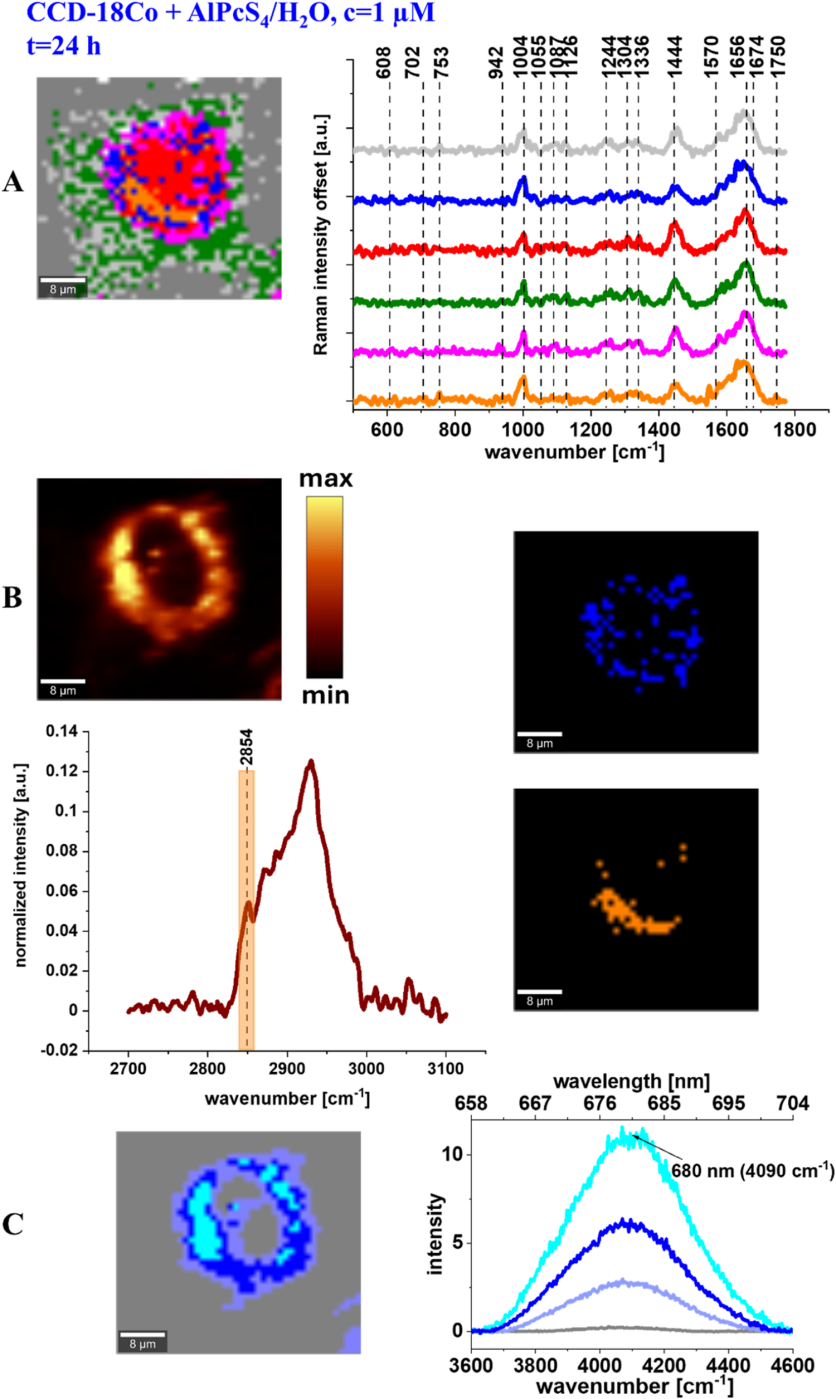
Raman imaging and mean Raman spectra of all organelles identified by CA: nucleus (red), mitochondria (magenta), ER (blue), LDs (orange), cytoplasm (green), cell membrane (light gray) (A), filter showing lipids distribution based on the Raman peak at 2854 cm^–1^, mean spectrum of the cell as a whole in the high-frequency range and the filter range marked in orange, maps of ER (blue) and LDs (orange) obtained based on CA (B), and Raman imaging of AlPcS_4_ distribution based on CA algorithm and fluorescence spectra of photosensitizer for normal human colon cells – CCD-18Co, for 24 h supplementation of the phthalocyanine (C), colors of the spectra correspond to colors of Raman maps.

A comparison of Figures 5 and 6 confirms that the main conclusion regarding the preferred localization of AlPcS_4_ in lipid-rich structures for short (30 min) incubation time also holds for 24 h of supplementation.

Moreover, the effect of incubation time is noticeable - the longer the incubation time, the more intense the photosensitizer fluorescence at the same acquisition time (see the signal intensity in panel C in Figures 5 and 6).

To test the ability of AlPcS_4_ to accumulate in human colon cancer cells, we performed experiments using the Caco-2 cell line. Similarly, as for normal colon cells (CCD-18Co), we launched the analysis from experiments for 30 min and 24 h of incubation time using AlPcS_4_, and for 30 min of incubation time using AlPcS_4_/DTAC solution. AlPcS_4_/DTAC solution was prepared according to the protocol described in ref 20. The results of all experiments are presented in Figure 7.

**Figure 7:**
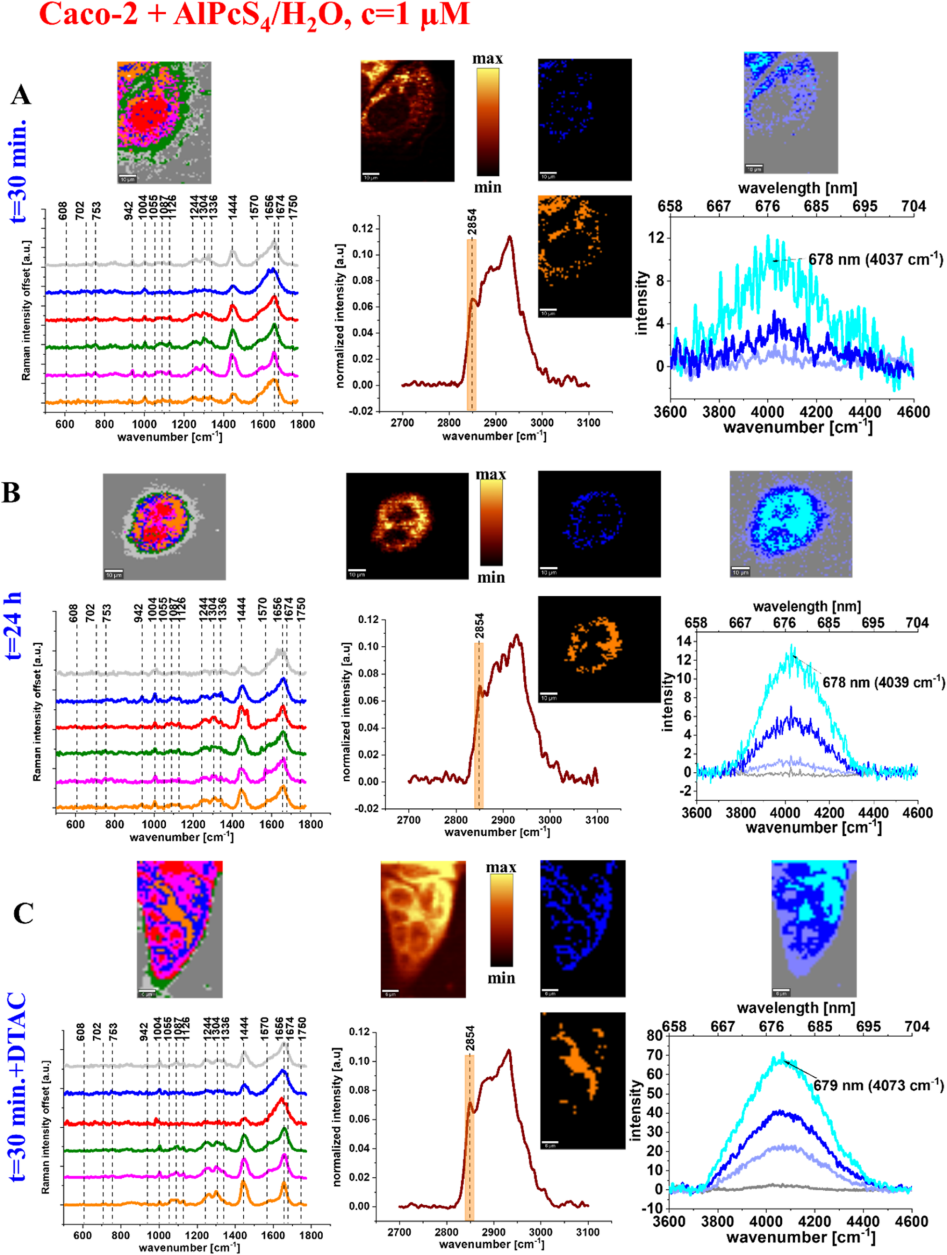
Raman imaging and mean Raman spectra of all organelles identified by CA: nucleus (red), mitochondria (magenta), ER (blue), LDs (orange), cytoplasm (green), cell membrane (light gray), filter showing lipids distribution based on Raman peak at 2854 cm^–1^, mean spectrum of the cell as a whole in the high-frequency range and the filter range marked in orange, maps of ER (blue) and LDs­(orange) obtained based on CA, and Raman imaging of AlPcS_4_ distribution based on CA algorithm and fluorescence spectra of photosensitizer (colors of the spectra correspond to colors of Raman maps) for 30 min of AlPcS_4_ incubation (A), for 24 h of AlPcS_4_ incubation (B) and 30 min of AlPcS_4_/DTAC incubation (C) for cancer human colon cells – Caco-2, colors of the spectra correspond to colors of clusters.

One can see from Figure 7 that, upon AlPcS_4_ supplementation for cancer human colon cells as for normal human colon cells, the photosensitizer is preferentially localized in ER and LDs. Moreover, the vibrational characteristic, based on the fingerprint region, is still available for normal cells. Figure 7 shows that, in contrast to cells without DTAC supplementation, where AlPcS_4_ localizes in ER and LD, in the case supplementation with DTAC the accumulation of AlPcS_4_ appears to be less selective. The intensities of fluorescence at 680 nm indicate that AlPcS_4_ localizes not only in ER and LD, but also in smaller concentrations in the nucleus. We have also found that the position of the fluorescence maximum of AlPcS_4_, determined by fitting with a Gaussian function, depends on the environment. In Caco-2 cells, the maximum is observed at 678 nm, while in CCD-18Co cells, it shifts to around 680 nm (cf. Figures 6C and 7A,B). For samples containing DTAC, the maximum is noticed at 677 nm (cf. Figure 7C). The difference in fluorescence maxima between normal and cancerous cells is an interesting aspect to consider when evaluating AlPcS_4_ as a potential diagnostic tool for distinguishing between normal and cancerous cells.

The intracellular concentration of AlPcS_4_ in normal and cancer human colon cells and the effect of DTAC addition for cancer human colon cells as a function of incubation time are summarized in Figure 8. Panel 8B shows expanded analysis of fluorescence intensity dependency from different incubation time intervals for cancer human colon cells with AlPcS_4_/DTAC micelles.

**Figure 8:**
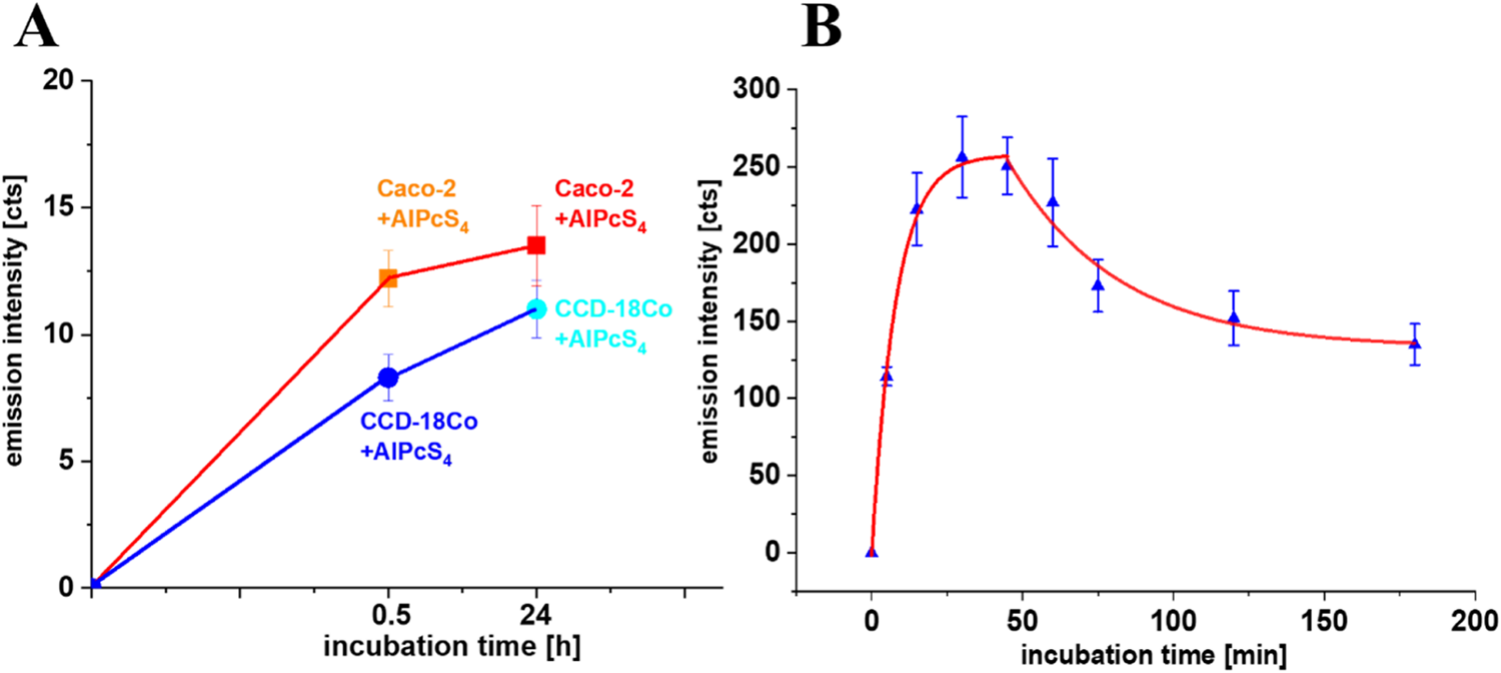
Incubation time effect for AlPcS_4_ in normal and cancer human colon cells (A) and the DTAC effect on cancer human colon cells (B) based on the fluorescence intensity of the photosensitizer. Error bars represent standard deviation (SD).

We observed that the intracellular concentration of DTAC, monitored via fluorescence intensity, initially increases in a monoexponential manner, with a time constant τ = 7.98 ± 0.72 min, reaching a maximum after approximately 45 min of incubation. This is followed by a gradual monoexponential decrease at longer incubation times, with a time constant τ = 36.23 ± 14.7 min (see Figure 8). For each incubation time point, fluorescence intensity was averaged over five individual cells. This pattern suggests that DTAC is rapidly taken up by the cells during the initial phase but is subsequently actively removed or degraded. The observed decline in fluorescence intensity may result from the decrease of osmotic potential inside the cell that initiates the process of cell disruption. These changes lead to the formation of hypertonic conditions inside the cell that cause increased membrane permeability and a cell swelling response, and may finally end in cell damage.

Summarizing the RS/RI measurements, we conclude that the effect of incubating human colon cells with AlPcS_4_ is time-dependent, and in cancerous human colon cells, it is effectively enhanced by the addition of DTAC. The time dependence of intracellular accumulation observed for AlPcS_4_ without DTAC agrees with previous studies published for other photosensitizers.^50^

Moreover, our experiments proved the preferred localization of photosensitizer in lipid structures (ER and LDs).

The literature has shown that the PC localization process in cellular substructures can be influenced by many factors, such as the central atom, substituents in the macrocycle, and the cell type (cancer type and/or stage of tumor development).^51,52^ In PDT, an appropriate photosensitizer should be capable of inducing the proper ROS concentration just after irradiation because ROS formation is a local effect, due to the short lifetime and minimal radius of radical action, therefore, the knowledge about the localization of the photosensitizer in cells and tissues is crucial for PDT efficiency.

Additionally, the intracellular localization of the PCs determines the mechanism of cell death.^53–55^ The localization of the photosensitizer in the cell membrane may cause necrosis as a result of the destruction of the membrane and loss of its integrity, in lysosomes or the ER, it may induce autophagy, and in mitochondria can lead to apoptosis.^53–59^ Our RS/RI measurements proved that the last two mechanisms refer to AlPcS_4_. The experiments also demonstrated that AlPcS_4_ preferentially accumulates in LDs. Fortunately, this photosensitizer behavior is also beneficial in PDT. Literature reports indicate that interactions between photosensitizers and LDs can lead to cellular iron-dependent lipid peroxidation. After irradiation, ferroptosis-mediated photodynamic therapy (PDT) can be initiated, resulting in high antitumor efficacy under both hypoxic and normoxic conditions.^60^ It should be noted that these effects are literature-based hypotheses and were not supported by data from our study.

Our experimental approach has been expanded to include femtosecond transient absorption experiments to examine the impact of the biological environment on the electronic relaxation time, a crucial parameter for the efficiency of a photosensitizer in PDT. First, we have recorded the time-resolved spectra of AlPcS_4_ in PBS (*c* = 10^–5^ M) which are shown in Figure 9A. These spectra include bands with maxima at 674 and 628 nm, both showing negative Δ*A*. These bands should be attributed to the ground state bleaching of AlPcS_4_. Another weaker band that shows a positive Δ*A*, with a maximum of around 570 nm, is also observed. Due to the positive sign of the Δ*A* signal in this spectral region, this band can be assigned to excited state absorption.

**Figure 9:**
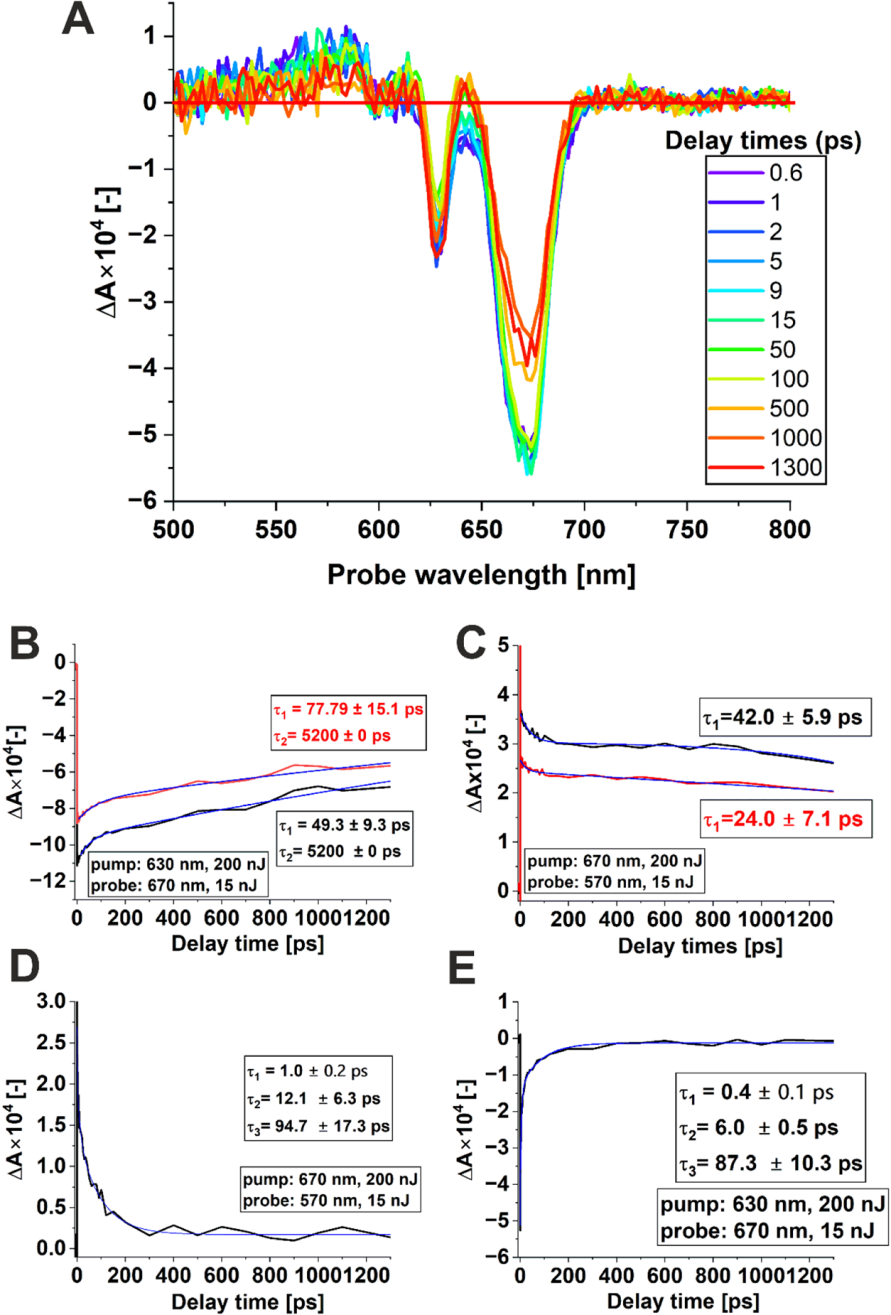
Time-resolved spectra of AlPcS_4_ in PBS following a 630 nm excitation, pulse duration ∼100 fs (A). The transient absorption kinetic traces of AlPcS_4_ (*c* = 10^–5^ M in PBS) alone (black line) and with the addition of CCD-18Co human colon cells (red line). The blue lines represent fittings with a two-exponential decay function. The values of τ_2_ were fixed at 5200 ps as they were determined from time-resolved fluorescence measurements. The pump and probe were set at 630 and 670 nm, respectively (B), The transient absorption kinetic traces of AlPcS_4_ (*c* = 10^–5^ M in PBS) alone (black line) and with the addition of CCD-18Co human colon cells (red line). The blue lines represent fittings with a two-exponential decay function. The pump and probe were set at 670 and 570 nm, respectively, pulse duration ∼100 fs (C), The transient absorption kinetic traces of AlPcS_4_ (*c* = 10^–5^ M) in H_2_O with the addition of DTAC (*c* = 16 mM). The blue lines represent fitting with a three-exponential decay function. The pump and probe were set at 630 and 670 nm, respectively (D), The transient absorption kinetic traces of AlPcS_4_ (*c* = 10^–5^ M) in H_2_O with the addition of DTAC (*c* = 16 mM). The blue lines represent fitting with a three-exponential decay function. The pump and probe were set at 670 and 570 nm (E).

To study the relaxation dynamics of AlPcS_4_ in the presence of cells and micelles, we have performed two-color transient absorption measurements. In these experiments, quasi-monochromatic probe wavelengths were used instead of a white light probe. Typically, two-color measurements provide a higher signal-to-noise (S/N) ratio, which is crucial for studying less homogeneous environments, such as solutions containing cells or micelles.

Figure 9B shows the transient absorption kinetic traces of AlPcS_4_ (*c* = 10^–5^ M in PBS) without (black line) and with the addition of CCD-18Co human colon cells (red line). The blue lines represent fittings of experimental data using a two-exponential decay function. The pump and probe were set at 630 and 670 nm, respectively.

The pump and probe were set at 630 and 670 nm based on the analysis of the absorption spectrum of AlPcS_4_ presented in Figure 10A. The 630 nm excites the weaker part of the Q-band AlPcS_4_ while the 670 nm probe the absorption near the maximum of the Q-band.

**Figure 10:**
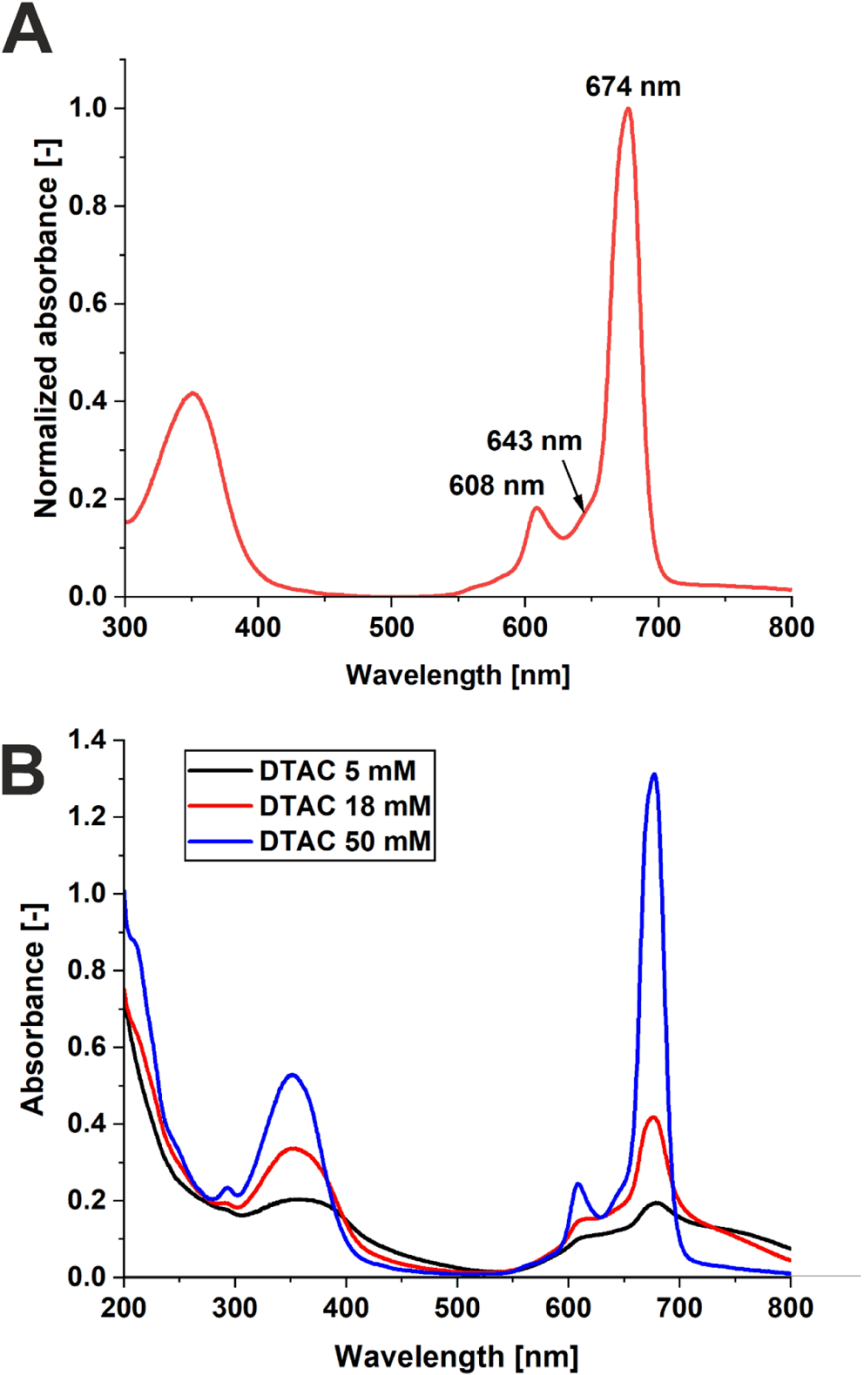
Normalized UV–vis absorption spectra of AlPcS_4_ at a concentration of 10^–5^ M in PBS (A), UV–vis absorption spectra of AlPcS_4_ at a concentration of 10^–4^ M with DTAC at varying concentrations (B).

One can see from Figure 10A that the Soret band and Q-band for AlPcS_4_ are observed. The B band features a maximum at 350 nm (S_0_→S_2_, a_2u_→e_g_, and b_2u_→e_g_ transitions) while the Q-band (S_0_ → S_1_, (a_1u_) → (e_g_) transition) in solution has a sharp maximum at 674 nm as well as weaker bands on the blue side with maxima at 643 and 607 nm.^61^ We have shown that the absorption bands are very similar in the concentration range of 10^–4^–10^–6^ M, indicating that AlPcS_4_ is dominated by a monomeric form in aqueous solution.^62^

The negative Δ*A* signals observed in Figure 9B should be assigned to ground-state bleaching since AlPcS_4_ strongly absorbs at 670 nm (see Figure 10A). The experimental data has been fitted with a two-exponential decay function that resulted in time constants of τ_1_ = 77.8 ± 15.1 ps for the sample without the addition of cells and τ_1_ = 49.3 ± 9.3 ps for the sample containing cells. In these fittings, the values of τ_2_ were fixed at 5200 ps, as they were precisely determined from time-resolved fluorescence measurements. The τ_1_ represents the vibrational relaxation of the AlPcS_4_ molecule, while the longer time constant, τ_2_, should be assigned to an electronic lifetime of S_1_. These results suggest that the presence of human colon cells does not significantly affect either the S_1_ lifetime or the vibrational relaxation time.

The time traces recorded for the pump at 670 nm and probe at 570 nm are presented in Figure 9C. Probing at 570 nm corresponds to the part of the ground state absorption spectrum of AlPcS_4_ at which the absorbance is small (see Figure 10). For this reason, the observed positive Δ*A* signals can be safely assigned to excited state absorption (S_1_→S*_n_* absorption). The two-exponential fittings feature a shorter time constant assigned to vibrational relaxation of 42.0 ± 5.9 ps for the sample without addition of cells, and 24.0 ± 7.1 ps for the sample containing cells. These values are of the same order of magnitude as those observed for the probe at 630 nm. On the other hand, for the probe at 570 nm, the long relaxation process is significantly longer than the corresponding one observed at 670 nm. For this reason, the determined time constants at 570 nm would not be reliable, as the corresponding Δ*A* signals significantly exceed the limitations of our delay line and are therefore not presented here. The presence of a very long relaxation time suggests a contribution from the triplet state of AlPcS_4_ (T_1_–T*_n_* transition). This is consistent with the previous literature findings for other phthalocyanines where the T_1_–T*_n_* was observed in 500–600 nm spectral region.^63,64^

We have also studied the impact of the presence of DTAC on the electronic relaxation of AlPcS_4_ in aqueous solution. As shown in Figure 10B, the addition of DTAC to the AlPcS_4_ solution in H_2_O alters the shape of the absorption spectrum compared to the homogeneous solution (without DTAC) at DTAC concentrations of 5 and 18 mM. For these DTAC concentrations, the appearance of a broad absorption band above 700 nm can be observed. This band is due to AlPcS_4_ aggregates. At a DTAC concentration of 50 mM (above the critical micelle concentration, CMC), the absorption spectrum of AlPcS_4_ closely resembles that of the homogeneous solution, confirming the absence of aggregation.

In general, in the case of phthalocyanines, the tendency to aggregation is strong and natural. Depending on the aggregate geometry, H-type aggregates (sandwich) and J-type aggregates (head to tail) can be distinguished, which, according to the Kasha excitonic model, strongly differ in terms of optical properties.^65^ In the case of AlPcS_4_, below the CMC for DTAC concentration of 22 mM at 25 °C^66^ this phthalocyanine shows monomer bands of reduced intensity and J-type aggregation (visible for bands above 700 nm). However, above CMC (*c* = 50 mM), J-type aggregation disappears.^20,67^

The impact of DTAC on the electronic properties of AlPcS_4_ can be further studied by femtosecond transient absorption spectroscopy. Figure 9D,E show the transient absorption signals for the aqueous AlPcS_4_ solution in a concentration of 16 mM with the addition of DTAC for the pump at 630 nm and probe at 670 nm, and for the pump at 670 nm and probe at 570 nm. The kinetics at both probe wavelengths are best fitted with three exponential functions. The determined time constants are 1.0 ± 0.2, 12.1 ± 6.3, and 94.7 ps for the probe at 570 nm, and 0.4 ± 0.1, 6.0 ± 0.5, and 87.3 ± 10.3 ps for the probe at 670 nm.

The most striking difference refers to the longest time constants, which take much shorter values compared to Δ*A* signals without the addition of DTAC or above CMC. This confirms the formation of J-type aggregates in aqueous solutions of AlPcS_4_ for concentrations of DTAC below CMC, which was reported by Correia et al.^20^ The strong interactions between units in aggregates facilitate efficient dissipation of electronic energy that entails a tremendous decrease in the electronic relaxation time. The two shorter time constants are also observed at both probe wavelengths. The time constants of several picoseconds (12.1 ± 6.3 ps for probe at 570 nm, and 6.0 ± 0.5 ps for probe at 670 nm). The time constants in this time scale can be assigned to vibrational relaxation. The shortest time constants (1.0 ± 0.2 ps for the probe at 570 nm and 0.4 ± 0.1 ps for the probe at 670 nm) are at the edge of the employed temporal resolution, resulting in considerable uncertainty about their values. Such a short time constant was previously observed for ZnPc and was assigned to the phase relaxation of S*_n_* states.^64,68^

The next step of our study was related to the analysis of the photochemical properties of AlPcS_4_ in a buffer solution with the addition of CCD-18Co human colon cells or Caco-2 colon cancer cells, which allows us to draw several conclusions.

The first rather unexpected observation is the small impact of the presence of cells on the absorption and fluorescence spectra of AlPcS_4_. It should be clearly emphasized that the analysis of the influence of the microenvironment on the spectral properties of AlPcS_4_ requires maintaining the similarity of the tested objects. The most important thing is to study objects with similar AlPcS_4_ absorbance. Figure 11 shows the spectrum of phthalocyanine in a buffer solution and the presence of two cell types. The value of the molar absorbance coefficient of AlPcS_4_ we determined is approximately 1.85 × 10^5^ M^–1^ cm^–1^ for the band with a maximum of 674 nm.

**Figure 11:**
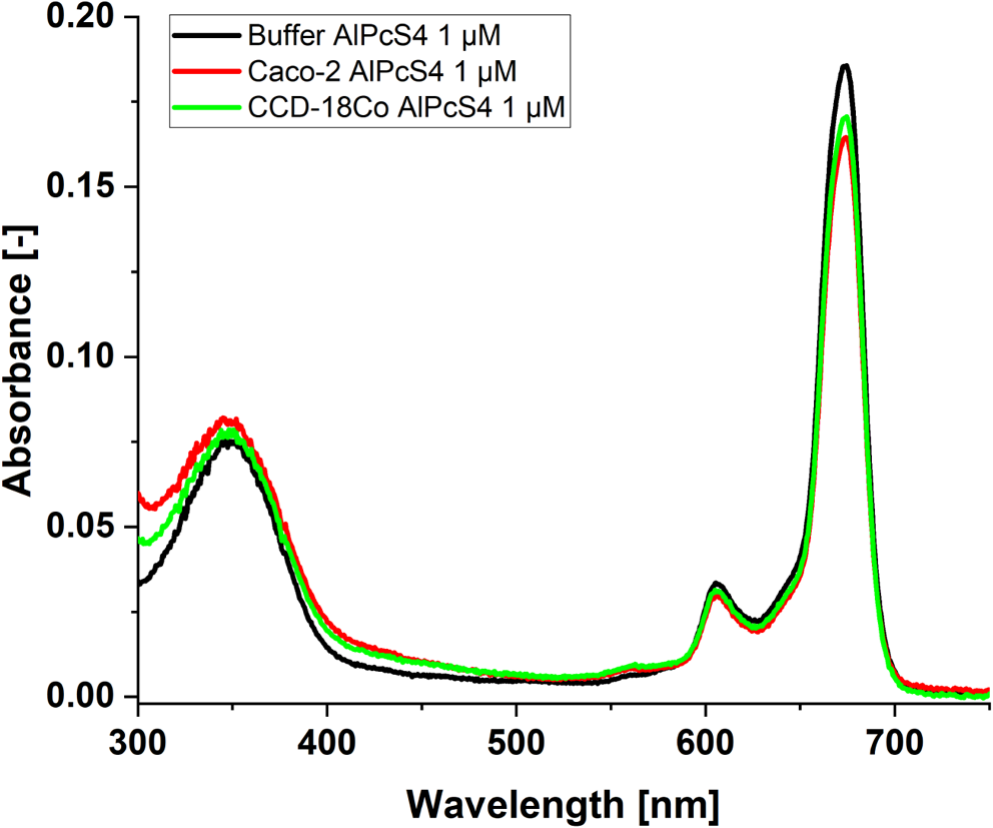
UV–vis absorbance spectrum of AlPcS_4_ in a buffer solution (black line) and in buffer solutions also containing cells: Caco-2 (red line) or CCD-18Co (green line). The concentration of AlPcS_4_ is equal to 1 μM.

The strong overlap of the long-wave absorption band with the fluorescence spectrum means that even at low phthalocyanine concentrations (of the order of several μM) corrections need to be made for inner filter effects. The Stokes shift for 1 μM AlPcS_4_ buffer solution is 0.0163 eV (the maximum of the absorption band is 674 nm, and the maximum of the fluorescence band is 680 nm). For a concentration of 10 μM, the maximum of the fluorescence spectrum of AlPcS_4_ is 684 nm. This 4 nm shift concerning the dilute solution is due to the inner filter effect (mainly absorption of fluorescence by phthalocyanine). Taking into account the inner filter effect makes the fluorescence spectra of phthalocyanine for concentrations of 1 and 10 μM identical. A detailed analysis of the emission spectra (excitation and emission) shows that the presence of cells does not strongly modify these spectra. The excitation spectra recorded at 690 nm perfectly match AlPcS_4_ in the entire spectral region of 280–680 nm for the use of buffer solution and solutions additionally containing healthy and cancer cells (Figure not shown). A very small difference between the fluorescence spectrum of AlPcS_4_ in the solution containing Caco-2 colon cancer cells is the higher intensity of the band in the spectral range of 700–760 nm compared to the analogous band for phthalocyanine in the solution with normal cells (see Figure 12).

**Figure 12:**
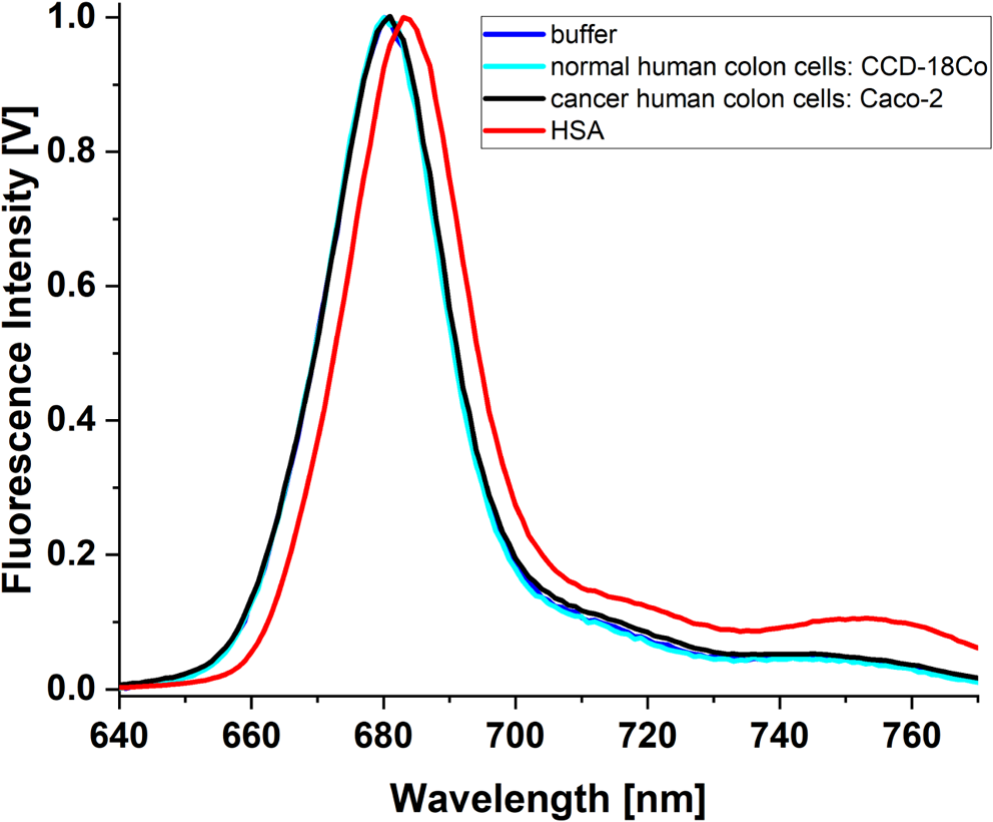
Fluorescence spectra of AlPcS_4_ (1 μM) recorded in buffer solution (blue line), in the presence: HSA (120 μM) - red line; normal human colon cells-cyan line, and cancer human colon cells - black line. Excitation wavelength: 610 nm. All spectra are corrected on inner filter effect and instrument response.

The fluorescence lifetimes of AlPcS_4_ in the buffer solution and the solution with cells measured by laser flash photolysis are very similar (5.2 vs 5.25 ns, respectively). The possibility of intracellular accumulation of AlPcS_4_ is confirmed by measurements of the triplet state of the photosensitizer. The transient difference spectrum (not shown) of AlPcS_4_ in the buffer solutions shows the absorption of the triplet centered at 490 nm, together with the Soret and Q-band ground state bleaches at 350 and 674 nm. The spectrum is consistent with that already published.^69^ We separately measured the lifetimes of the excited triplet state of AlPcS_4_ in a buffer solution, a buffer containing HSA, or both kinds of cells in vacuum-deaerated solutions. Results are presented in Figure 13. These times are different; the shortest recorded in the buffer solution is 407 μs, for AlPcS_4_ in the presence of cells, 460 μs, while inside HSA, the triplet lifetime is 750 μs.

**Figure 13:**
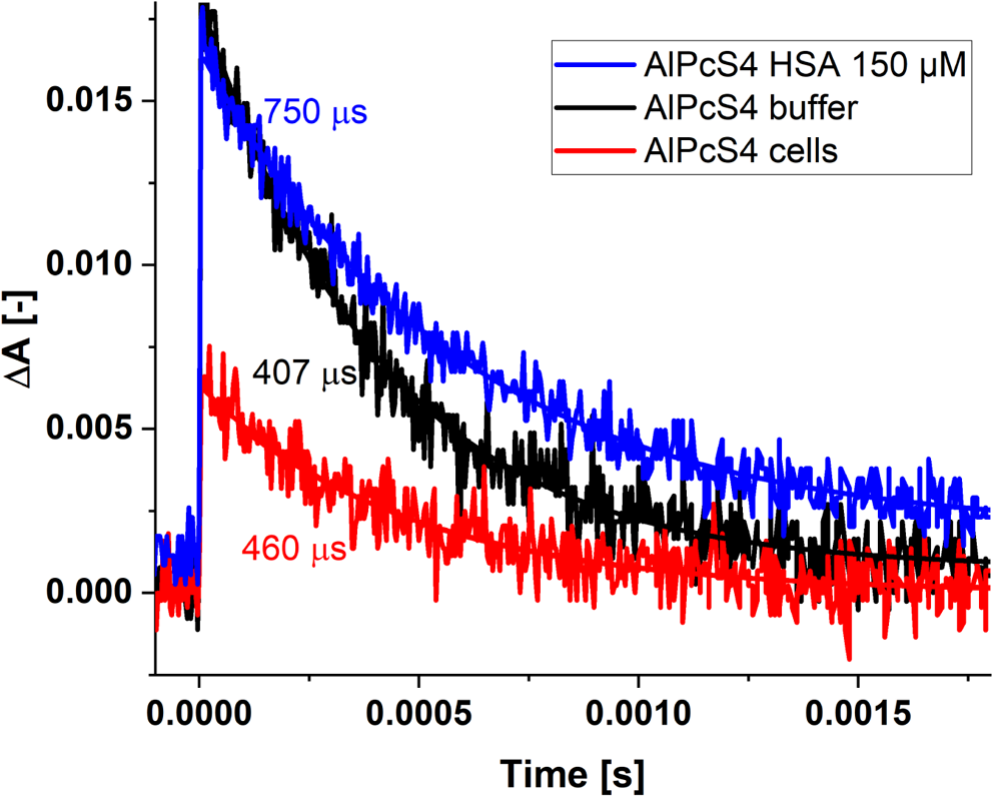
Time profiles recorded at 490 nm after excitation at 337 nm (pulse duration 800 ps, energy 1.9 mJ) for AlPcS_4_ (5 μM) in buffer solution (black noisy line), in the presence of HSA (150 μM) (blue noisy line), and in the presence of human colon cells (red noisy line, AlPcS_4_ (1 μM)). The excited triplet state decay in each case is well described by a single exponential function (smooth lines). The numbers in the figure indicate the decay times of the AlPcS_4_ triplet state in a given solution.

It has been proposed^69^ that AlPcS_4_ binds to the protein, where it is protected from the aqueous phase. The longer lifetime of the triplet state in the presence of cells (identical for Caco-2 colon cancer cells and CCD-18Co cells) than observed in the buffer solution is strong evidence for the interaction of phthalocyanine with cells. This enables the production of singlet oxygen using AlPcS_4_ as a photosensitizer in the photodynamic method of treating colorectal cancer.

Summarizing, recent advancements in Photodynamic Therapy (PDT) and spectroscopic techniques have significantly enhanced our ability to diagnose and treat various diseases, particularly cancer. PDT, a treatment modality that utilizes photosensitizers activated by light to induce cytotoxicity in targeted tissues, has seen considerable improvements in both photosensitizer development and light delivery systems. New generation photosensitizers, including those with enhanced tissue penetration and reduced dark toxicity, offer better selectivity for tumor tissues while minimizing side effects. These advancements have been complemented by innovations in spectroscopy, which have allowed for more precise monitoring of PDT treatments in real-time.

We have proved that spectroscopic techniques, such as Raman spectroscopy, fluorescence spectroscopy, provide valuable insights into the dynamics of photosensitizer distribution and activation, as well as cellular responses to PDT. Raman spectroscopy, for example, can noninvasively map the molecular composition of cells and track changes induced by PDT, offering a unique way to evaluate the effectiveness of treatment at the cellular and subcellular levels. Additionally, the development of dual-modality imaging systems combining PDT with spectroscopic methods has enhanced the ability to visualize treatment progress, offering a more comprehensive understanding of the underlying biochemical processes.^70–72^

When comparing emerging photosensitizers, recent studies have focused on the development of compounds with improved properties, such as enhanced singlet oxygen generation, higher stability, and better solubility in physiological environments. Some of these new photosensitizers, such as porphyrins (e.g., Verteporfin), phthalocyanines (e.g., AlPcS_4_), and chlorins (e.g., Temoporfin), offer more effective targeting of hypoxic tumor regions, which are often resistant to traditional PDT treatments.^73,74^ In comparison to older generations of photosensitizers, these new compounds demonstrate superior tumor targeting and deeper tissue penetration, making them suitable for a wider range of clinical applications. For instance, Verteporfin has been successfully used for the treatment of macular degeneration, while AlPcS_4_ has shown potential for effective PDT in the treatment of brain tumors due to its ability to penetrate deeper tissues.^75,76^ However, challenges remain in optimizing the balance between photosensitizer efficacy and minimizing off-target effects, such as skin photosensitivity. Additionally, the integration of spectroscopic techniques with PDT requires further refinement to achieve real-time, accurate monitoring during treatment. As new photosensitizers continue to emerge and spectroscopic methods evolve, the combination of these technologies holds the potential to improve both the precision and outcomes of PDT, moving closer to personalized, effective treatments for various malignancies.^73,74,77–79^

## Conclusions

We investigated the spectroscopic properties of AlPcS_4_, a promising photosensitizer for photodynamic therapy (PDT), using Raman imaging, electronic absorption spectroscopy, fluorescence spectroscopy, and transient absorption spectroscopy. These techniques were employed to determine the distribution of the photosensitizer in human colon tissue and to study its dynamic behavior in aqueous solutions, including cell-containing environments.

We have found that, following AlPcS_4_ supplementation in human colon cancer cells, the photosensitizer preferentially localizes to the endoplasmic reticulum and lipid droplets, as observed in normal cells. We observed that the addition of DTAC significantly increases the permeability of cell membranes to AlPcS_4_, resulting in a higher concentration of the photosensitizer inside the cell, as observed by increased intensity of fluorescence around 679 nm, despite only 30 min of incubation. We observed that the intracellular concentration of DTAC initially increases in a monoexponential manner, with a time constant τ = 7.98 ± 0.72 min, reaching a maximum after approximately 45 min of incubation, which is followed by a gradual monoexponential decrease at longer incubation times, with a time constant τ = 36.23 ± 14.7 min. This pattern suggests that DTAC is rapidly taken up by cells but later removed or degraded, with the decline in fluorescence likely linked to osmotic imbalance, increased membrane permeability, cell swelling, and eventual cell damage.

The steady-state absorption and fluorescence spectra, as well as the singlet-state lifetimes of AlPcS_4_ in buffer or aqueous solution, are nearly identical to those in samples containing cells, suggesting minimal interaction between the photosensitizer and cells. However, the extended triplet-state lifetime observed in the presence of both Caco-2 and CCD-18Co cells (460 μs), compared to buffer alone (407 μs), provides clear evidence of interaction between AlPcS_4_ and the cells. A long triplet lifetime is generally favorable for singlet oxygen generation in photodynamic therapy, as it increases the probability of energy transfer to molecular oxygen. However, the triplet state must also possess sufficient energy and be accessible to oxygen for efficient ^1^O_2_ production. However, in this manuscript, no direct quantification of ROS was performed, and further research is needed.

While AlPcS_4_ does not tend to aggregate in aqueous solution, the addition of the DTAC at concentrations below the CMC to aqueous AlPcS_4_ solution led to the formation of J-type aggregates, as evidenced by the broad band above 700 nm in the UV–vis absorption spectrum and a significant decrease in the S_1_ lifetime (from 5.2 ns to about 90 ps) observed in femtosecond transient absorption experiments. This effect was observed only in aqueous solutions of AlPcS_4_ and not in PBS solutions.
